# Cartas al editor

**Published:** 2026-08-20

**Authors:** Valeria Alejandra Chávez, Ariel Nicolás Landa, Johan Alexander Azañero

**Affiliations:** 1 Facultad de Medicina, Universidad Nacional Federico Villarreal, Lima, Perú; Sociedad Científica de Estudiantes de Medicina Villarrealinos, Lima, Perú. Universidad Nacional Federico Villarreal Facultad de Medicina Universidad Nacional Federico Villarreal Lima Peru; 2 Facultad de Medicina, Universidad Nacional Federico Villarreal, Lima, Perú; Sociedad Científica de Estudiantes de Medicina Villarrealinos, Lima, Perú. Universidad Nacional Federico Villarreal Facultad de Medicina Universidad Nacional Federico Villarreal Lima Peru; 3 Facultad de Medicina, Universidad Nacional Federico Villarreal, Lima, Perú; Departamento de Medicina Universidad Nacional Federico Villarreal Facultad de Medicina Universidad Nacional Federico Villarreal Lima Peru

Lima, 24 de mayo del 2026

Señores

Revista *Biomédica*

Instituto Nacional de Salud 

Bogotá, D. C., Colombia

Estimados señores:

Hemos analizado con gran interés el artículo de Sánchez-Amador y colaboradores titulado "Enfermedades infecciosas respiratorias en pacientes de unidades de cuidados intensivos en dos hospitales de Guayaquil, Ecuador, 2024" ^1^, el cual representa un esfuerzo valioso para caracterizar la ecología microbiana y los perfiles de resistencia en el entorno crítico. Aplaudimos la iniciativa de integrar la microbiología convencional con plataformas de diagnóstico molecular rápido, una estrategia alineada con la búsqueda de la medicina de precisión ^1^^,^^2^. No obstante, desde la perspectiva de la epidemiología clínica y el rigor metodológico, existen observaciones de fondo que matizan de forma importante la interpretación y validez de sus hallazgos.

En primer lugar, salta a la vista una acentuada falta de homogeneidad en el criterio de selección de la muestra. Pese a que el título compromete al lector con enfermedades infecciosas respiratorias, el corpus de resultados exhibe diagnósticos definitivos completamente disímiles como septicemias, derrames pleurales e inclusive tumores benignos ^1^. Mezclar infecciones sistémicas primarias con afecciones estrictamente parenquimatosas fragmenta la coherencia del análisis. A este problema se suma un claro sesgo de información: no queda claro si los denominadores utilizados corresponden a pacientes, a muestras o a aislamientos individuales. Esta ambigüedad operativa debilita de entrada la reproducibilidad y la comparación externa de los datos.

En segundo lugar, se detecta desfase entre el diseño metodológico adoptado y la interpretación clínica de los eventos. Siguiendo las pautas metodológicas clásicas, los estudios transversales capturan la información en un punto único en el tiempo, lo que anula cualquier intento de establecer direccionalidad temporal ^3^. Por tanto, el uso de terminología evolutiva en el artículo -al sugerir que los pacientes «evolucionaron a neumonía intrahospitalaria» o catalogar a los virus como «desencadenantes»- excede los límites inferenciales del diseño ^3^. Para sustentar tales afirmaciones causales se requería un seguimiento longitudinal, complementado con variables de gravedad clínica (escalas SOFA o APACHE II) indispensables para contextualizar el verdadero pronóstico de la cohorte ^4^.

Un punto crítico ausente en la investigación es la delimitación entre la colonización microbiológica de la vía aérea y la infección pulmonar verdadera. En la unidad de cuidados intensivos, el aislamiento de microorganismos como *Candida* spp. o *Acinetobacter baumannii* en secreciones respiratorias a menudo representa persistencia biológica saprofítica sin repercusión patológica ^5^^,^^6^. *Los autores omitieron el uso de criterios clínico-radiológicos validados, como el Clinical Pulmonary Infection Score,* para aseverar que los cultivos positivos correspondían a cuadros de neumonía activa ^7^.

Asimismo, el estudio procesó cultivos como positivos bajo un umbral genérico y excesivamente bajo (de 10^3^ o 10^4^ UFC/ml), ignorando la naturaleza de la muestra ^1^. Consensos internacionales vigentes, como los de la *American Thoracic Society* (ATS) y la *Infectious Diseases Society of America* (IDSA), exigen puntos de corte drásticamente mayores para salvaguardar la especificidad diagnóstica: ≥ 10^4^ UFC/ml para lavado broncoalveolar, ≥ 10^5^ UFC/ml para aspirado traqueal y ≥ 10^3^ UFC/ml para cepillado bronquial ^8^^,^^9^. El empleo de umbrales laxos induce una sobredetección de agentes colonizadores, inflando artificialmente las tasas de neumonía y coinfecciones reportadas.

Por otro lado, respecto a la tecnología FilmArray™, si bien su velocidad revoluciona el manejo inicial, los paneles de PCR múltiple amplifican el material genético de microorganismos no viables o remanentes de procesos ya resueltos ^10^. Esto produce falsos positivos moleculares si no se filtra cada resultado bajo un tamiz clínico estricto. Además, el texto incurre en una confusión común entre la detección genotípica y la resistencia fenotípica real; el portar un gen de resistencia no garantiza su transcripción ni su expresión funcional, debido a dinámicas de regulación o a la presencia de pseudogenes, razón por la cual el antibiograma convencional se mantiene como la prueba de referencia irremplazable para consolidar la terapia definitiva ^10^.

Finalmente, el tamaño limitado de la muestra (n = 56) imposibilita esbozar conclusiones epidemiológicas extrapolables. Al carecer de pruebas de estadística inferencial, los datos quedan relegados a una descripción local propensa a variaciones por el puro azar. Asimismo, la discusión se adentra en explicaciones ecológicas basadas en el clima sin un modelado formal, cayendo en la clásica falacia ecológica al correlacionar datos macroambientales de humedad con respuestas fisiopatológicas individuales dentro de un entorno altamente controlado como la unidad de cuidados intensivos.

Consideramos que estas anotaciones, lejos de demeritar el esfuerzo de los autores, son indispensables para robustecer los estándares de la investigación en medicina crítica y garantizar que la implementación de tecnologías de punta esté respaldada por un rigor metodológico intachable.
