# Supplementary material for: Cartas al editor
Source: Biomedica. 2026 Aug 20;46(3):504–6. [Article in Spanish] (PMC13649250)
Supplement: Related texts

# Reply: Cartas al editor

Guayaquil, 29 de mayo del 2026

Señores editores

Revista *Biomédica*

Instituto Nacional de Salud

Bogotá, D. C., Colombia

Estimados señores:

En respuesta a la carta al editor titulada: «Diagnóstico molecular rápido y ecología microbiana en cuidados intensivos: precisiones metodológicas indispensables», se argumenta a continuación.

Agradecemos los comentarios realizados a nuestro manuscrito, pues permiten precisar aspectos metodológicos relevantes para la interpretación de los hallazgos.

En primer lugar, aclaramos que el estudio incluyó pacientes hospitalizados en la unidad de cuidados intensivos con sospecha clínica o diagnóstico presuntivo o definitivo de enfermedad respiratoria, a partir de muestras respiratorias procesadas por microbiología convencional y biología molecular. Por ello, algunos diagnósticos de ingreso o comorbilidades no respiratorias coexistieron con el evento respiratorio evaluado. No obstante, reconocemos que la redacción pudo generar ambigüedad y en futuras versiones se precisará con mayor claridad la unidad de análisis: pacientes, muestras y aislamientos.

Respecto al diseño transversal prospectivo, coincidimos en que no permite establecer causalidad ni direccionalidad temporal. En consecuencia, expresiones como «evolucionaron a neumonía intrahospitalaria» o «desencadenantes» deben interpretarse como asociaciones clínicas observadas y no como inferencias causales. Aceptamos que una redacción más prudente sería: «(...) presentaron diagnóstico de neumonía intrahospitalaria durante la hospitalización (...)» y «(...) se asociaron con coinfecciones bacterianas (...)».

En relación con la diferenciación entre colonización e infección, compartimos que este es un desafío central en pacientes críticos. La interpretación microbiológica fue realizada dentro del contexto clínico de cada paciente y complementada con cultivos convencionales, pruebas de sensibilidad antimicrobiana y el panel molecular FilmArray™. Sin embargo, reconocemos que la incorporación explícita de criterios clínico-radiológicos, como CPIS, SOFA, APACHE II o los criterios de la *American Thoracic Society* (ATS) y la *Infectious Diseases Society of America* (IDSA) para neumonía hospitalaria o neumonía asociada a repiración mecánica asistida, habría fortalecido la clasificación diagnóstica.

Sobre los puntos de corte microbiológicos, aceptamos que estos deben ser descritos según el tipo de muestra respiratoria y de acuerdo con los estándares internacionales. En futuros artículos sobre este tema revisaremos la redacción metodológica para evitar interpretaciones erróneas y diferenciar claramente los cultivos semicuantitativos, los resultados moleculares y la interpretación clínica de positividad.

Respecto al FilmArray™, coincidimos en que la detección molecular no sustituye al cultivo ni al antibiograma fenotípico. En nuestro estudio, la plataforma fue utilizada como herramienta complementaria para identificación rápida de agentes patógenos y genes de resistencia, no como reemplazo de las pruebas convencionales.

Finalmente, reconocemos que el tamaño muestral de 56 pacientes y el período de seis meses limitan la extrapolación epidemiológica. Nuestro objetivo fue descriptivo y exploratorio, orientado a generar información local sobre agentes respiratorios y genes de resistencia en las unidades de cuidados intensivos.

Agradecemos nuevamente las observaciones recibidas, las cuales contribuirán a mejorar la precisión metodológica del manuscrito y a fortalecer la interpretación clínica de los resultados.

Agradezco el interés ante la presente, esperando una respuesta optimista del mismo artículo publicado.

Francisco Sánchez A., Mg. Biol.
